# Knowledge, Attitude, and Practice Regarding Vision and Eye Screening of Preschool Children Among Primary Health Center Staff in the Qassim Region, Saudi Arabia

**DOI:** 10.7759/cureus.52743

**Published:** 2024-01-22

**Authors:** Dora H AlHarkan, Nawaf S AlRubaysh, Mohammad I Aldekhail, Saleh A Alayidi, Meshal S Alashgar, Faisal F Almishali

**Affiliations:** 1 Ophthalmology, Medical College, Qassim University, Buraydah, SAU; 2 General Practitioner, King Fahad Specialist Hospital, Buraydah, SAU; 3 General Practitioner, Buraydah Central Hospital, Buraydah, SAU

**Keywords:** primary health center, childhood blindness, practice, attitude, knowledge, vision and eye screening

## Abstract

Purpose

To study the knowledge, attitude, and practice (KAP) regarding vision and eye screening of preschool children among primary health center (PHC) staff in Qassim, Saudi Arabia.

Methods

A survey of PHC staff was conducted in 2023. The questionnaire included knowledge (10), attitude (five), and practice (five)-related questions associated with preschool vision and eye screening. A five-graded Likert scale was used for responses. Cronbach’s alpha score of the questionnaire was 0.776. The KAP score was correlated with the demographic variables of participants. The current and desired sources of information were also collected.

Results

We surveyed 101 health staff (66 doctors and 35 nurses). The median (interquartile range) knowledge, attitude, and practice scores of participants were 4.1 (3.8; 4.3), 4.2 (4.0; 4.6), and 3.6 (3.0; 4.0), respectively. The doctors had better knowledge (Mann-Whitney U test (MW), P = 0.016) and attitude (MW, P = 0.019) than the nurses. Staff above 40 years had better knowledge (Kruskal-Wallis H test (KW), P = 0.035), attitude (KW, P = 0.017), and practice (KW, P < 0.001). The primary source of information about preschool vision screening was their medical education (51%). Other sources were eye care professionals (11.9%), Google and computers (12.9%), and social media (14.9%). Their preferred sources of information were medical journals (25.7%), eyecare training (22.8%), and eye professionals (33.7%).

Conclusions

Knowledge and attitude for eye and vision screening of preschool children was high, but practices were less among PHC staff. Providing information through their preferred mode could further strengthen eye care for preschool children.

## Introduction

By integrating primary eye care within primary health care, a comprehensive and systematic approach can be developed to prevent and treat childhood blindness [1]. Primary health center (PHC) staff can detect eye problems in the early stages, refer children to eye care professionals, counsel parents to improve compliance with advice related to eye health, and even undertake opportunistic vision and eye screening at children’s visits for immunization or treatment for general health issues [2,3]. Eye care that is integrated and coordinated in primary-level health facilities will be enabled by training primary health care workers and allied personnel to undertake such tasks [4].

The World Health Organization prioritized childhood blindness in the VISION 2020 initiative to address avoidable blindness and advised member countries to apply strategies for early detection and referral systems as part of primary eye care integrated with child health care programs [5,6].

The Ministry of Health of the Kingdom of Saudi Arabia activated school-based screening health care programs, including vision screening of first- and fourth-grade primary school children through health centers in 2021 [7]. To the best of our knowledge, eye and vision screening of three- to five-year-old children, as recommended by the American Pediatric Association, has not yet been implemented in the Kingdom of Saudi Arabia [8]. A study that evaluated the vision screening programs of 46 countries revealed wide variation in age-groups and methods of testing the vision of children in different countries [9].

In particular, the strategy for preschool vision and eye screening requires the support of trained primary health doctors and nurses. Therefore, it is essential to determine preschool children’s level of awareness and attitude as well as current practices regarding vision and eye screening. The literature mentions variable levels of knowledge, attitude, and practice (KAP) among primary eye care staff, particularly pediatricians, PHC doctors, and nurses [10-13]. Researchers in different provinces of Saudi Arabia have noted a low level of awareness of strabismus and amblyopia among pediatricians and family physicians [14,15].

The Qassim Province of Saudi Arabia has 155 PHCs in the Ministry of Health, and approximately 466 doctors and 990 nurses provide child health care at these PHCs [16]. These health care workers were not formally trained in providing primary eye care or undertaking vision and eye screening of children. To generate evidence to determine the need for preschool vision screening (PVS) and develop the capacity of stakeholders, including primary health care providers, it is essential to identify the current level of awareness, attitudes, and practices of eye care for children.

We examine primary health care staff’s perceived level and determinants of KAP for preschool vision and eye screening in Qassim.

## Materials and methods

Study overview

The doctors and nurses of all the PHCs in Qassim Province were informed about the survey. The study investigators visited selected health centers and recruited participants randomly from people who were present at PHCs at the time of the visit.

Ethical consideration

The ethical and research committee approved this study (607/44/12460 dated 13/3/2023). Written informed consent was obtained from the participants for the survey. Those who declined to participate were excluded from the study. This study strictly abided by the tenets of the Helsinki Declaration. The personal identity of the participants was kept confidential.

Study criteria

The study population was around 1,500 doctors and nurses working in the PHCs providing child health care in the Qassim region. Of the 155 PHCs, four randomly selected PHCs were visited by the field investigators. Those on duty who consented to participate were included in the survey. The PHCs were visited at two different shifts on different days to increase the coverage of the survey participants.

Study procedures

A Google Form prepared in consultation with pediatric ophthalmologists, PHC doctors, and epidemiologists was loaded on tablets for participants to provide their responses.

Demographic information included gender, age-group, city of the PHC, and the category of health staff (doctor or nurse).

Assessments

There were 10 questions on knowledge, five questions on attitudes, and five questions on practices related to preschool vision and eye screening. Five-point Likert scale responses included “I fully disagree,” “I disagree,” “not sure,” “I agree,” and “I fully agree.” The questions were in the English language (Appendix Table 3). However, the investigators had an Arabic translation ready if a participant did not understand a question in English and needed clarification.

The questionnaire was translated into Arabic, and then reverse translation was performed by scholars not involved in the survey. The wording of the questions was kept simple for easy understanding by PHC staff. Ten health staff members were asked to complete the form in the pilot study. The pilot data were analyzed to study the reliability of the questions, and Cronbach’s alpha of 0.776 was noted. The responses of 10 participants of the pilot study were entered into IBM SPSS Statistics, version 25.0 (IBM Corp., Armonk, NY). Using the reliability analysis tool under the scale analysis, we grouped responses to KAP questions into two groups and then estimated Cronbach’s alpha value. A value of more than 0.75 is considered very good. The Likert scale analysis was performed in line with the internationally recommended method for health studies [17]. The five levels of Likert scale responses were given a score of 1-5.

Sample size calculation

To calculate the sample size to represent 1,500 doctors and nurses at 155 PHCs in the Ministry of Health in Qassim [16], we assumed that 68% of participants had good knowledge about refractive error in children, as noted in the Jordan study [11]. To achieve a 95% confidence interval, a 10% acceptable error margin (the level of KAP could have a variation of 10% among surveyed participants), and a clustering effect of 1.2 (there could be a variation of KAP based on PHC that was randomly selected), we needed to randomly select 96 PHC staff. To compensate for incomplete responses, we increased the sample to 100. We used the sample size calculation of Open-Epi software [18].

Statistical analysis

The data from the Google Form were transferred into a spreadsheet in the IBM SPSS Statistics, version 25.0 (IBM Corp., Armonk, NY). The median response score for the question knowledge group was calculated to determine the overall knowledge score. A similar exercise was performed for the attitude and practice groups of questions. We plotted graphs to visualize the 5-point Likert scale responses on KAP. We used a nonparametric method to compare the overall knowledge response score in a subgroup. The Mann-Whitney U test was used for two subgroups, and Z- and P-values were noted. For more than two subgroups, we used the Kruskal-Wallis H test. Chi-square and two-sided P-values were estimated. A P-value of <0.05 was considered statistically significant.

## Results

We surveyed 101 PHC staff in the study area. There were 66 doctors (65.3%) and 35 nurses (34.7%), including 55 males (54.5%) and 46 females (45.5%). The participants’ age-groups were 20-29 years (46; 45.6%), 30-39 years (30; 29.7%), and above 40 years (25; 24.8%). The participants were from the PHCs of four cities: Buraidah (59; 58.4%), Unaizah (21; 20.8%), Al Rass (8; 7.9%), and Al Bukayriyah (13; 12.9%).

**Figure 1:**
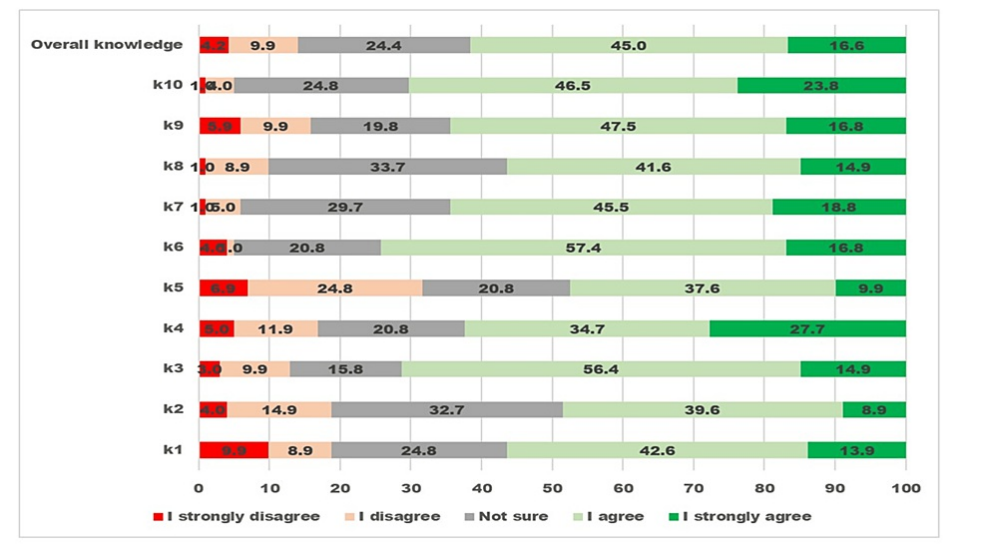
Visualizing the five-graded Likert scale responses to questions about vision and eye screening of preschool children related to “knowledge” by primary health staff The X-axis displays the percentage proportion of participants giving a grade of response. The Y-axis displays 10 knowledge-related questions and one overall knowledge.

The responses to 10 knowledge-related questions and overall knowledge about preschool vision and eye screening among PHC staff are presented in Figure 1.

More than 60% of participants agreed with the correct knowledge statements. The item “<5-year-old children’s eye problems can be found only with vision screening” had the lowest rate of agreement, while the item “<5-year-old children with refractive error should wear spectacles” had the highest agreement. For all questions, one in five participants was “not sure.”

**Figure 2:**
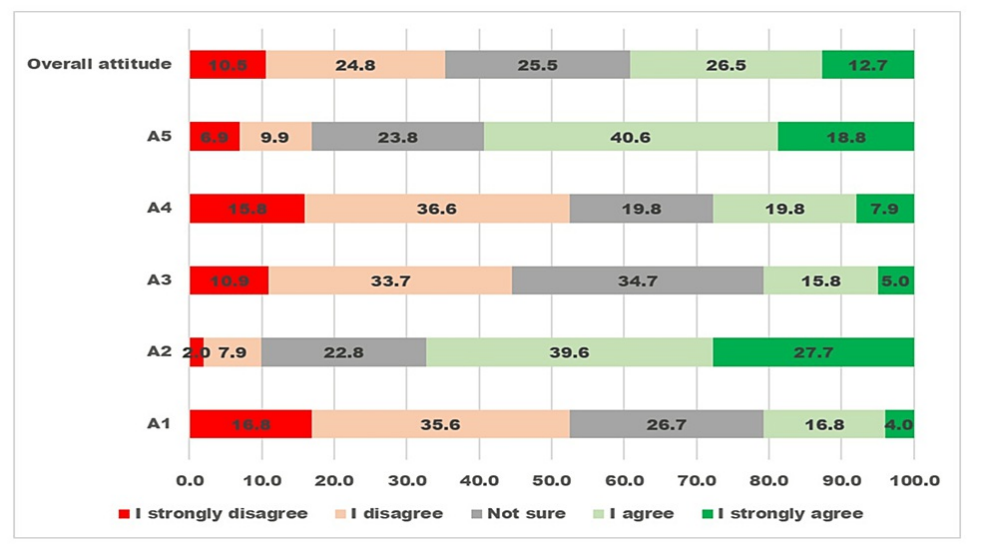
Visualizing the five-graded Likert scale responses to questions about vision and eye screening of preschool children related to “attitude” by primary health staff The X-axis displays the percentage proportion of participants giving a grade of response. The Y-axis displays five attitude-related questions and one overall attitude.

The responses to five attitude-related questions and overall attitudes about preschool vision and eye screening among PHC staff are given in Figure 2.

Nearly four in 10 participants had an overall positive attitude toward preschool vision and eye screening. The questions “I believe spectacles should not be used by young children as it increases the need for spectacles” and “I believe children are negatively psychologically affected and therefore should not be given spectacles” had responses suggestive of a high proportion of negative attitudes.

**Figure 3:**
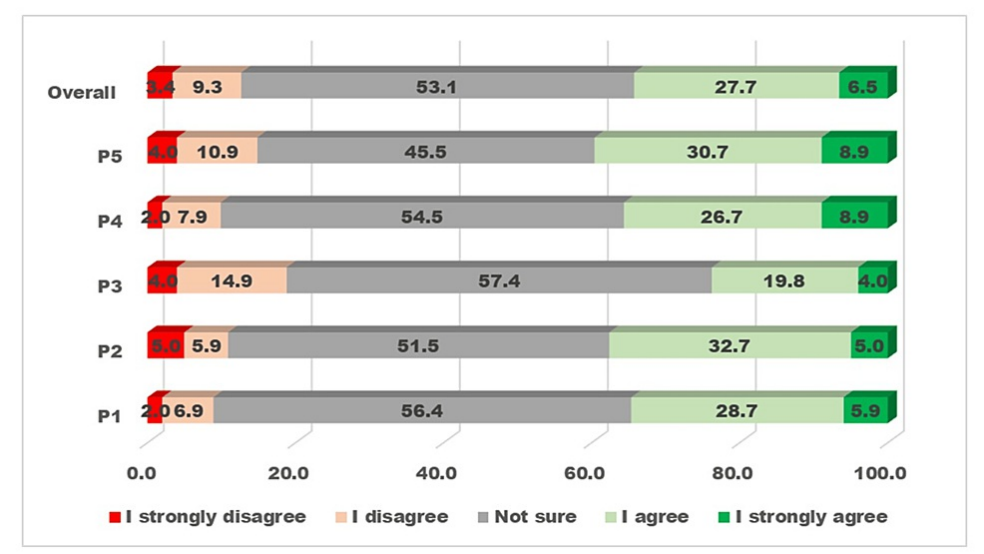
Visualizing the five-graded Likert scale responses to questions about vision and eye screening of preschool children related to “practice” by primary health staff The X-axis displays the percentage proportion of participants giving a grade of response. The Y-axis displays five practice-related questions and one overall practice.

The responses to five practice-related questions and overall practices related to preschool vision and eye screening among PHC staff are presented in Figure 3.

One-third of participants had responses suggesting good practices for vision and eye screening for preschoolers. More than half of the participants were not sure about the practice-related questions.

The KAP scores of the participants and subgroups are given in Table 1.

**Table 1:** Knowledge, attitude, and practice related to vision and eye screening of preschool children among PHC staff in Qassim, Saudi Arabia *For two subgroups, the P-value is based on the Mann-Whitney U test. Meanwhile, for more than two subgroups, the P-value is based on the Kruskal-Wallis H test. IQR, inter quartile range; PHC, primary health center

		Knowledge		Attitude		Practice	
		Median (IQR)	*P-value	Median (IQR)	P-value	Median (IQR)	P-value
All PHC staff		4.1 (3.8-4.3)	-	4.2 (4.0-4.6)	-	3.6 (3.0-4.0)	-
Gender	Male	4.1 (3.8-4.3)	0.88	4.2 (4.0-4.6)	0.565	3.6 (3.0-4.0)	0.821
	Female	4.1 (3.78-4.3)		4.2 (3.95-4.45)		3.6 (3.0-4.0)	
Age-group	20-29	4.0 (3.7-4.3)	0.034	4.2 (3.75-4.4)	0.017	3.0 (3.0-3.65)	<0.001
	30-39	4.0 (3.8-4.2)		4.2 (3.95-4.6)		3.8 (3.0-4.0)	
	40+	4.3 (4.1-4.35)		4.4 (4.02-4.8)		4.0 (3.0-4.4)	
Staff category	Doctor	4.15 (3.9-4.3)	0.016	4.2 (4.0-4.6)	0.019	3.8 (3.0-4.1)	0.107
	Nurse	4.0 (3.5-4.2)		4.0 (3.0-4.4)		3.0 (3.0-4.0)	
City	Buraidah	4.2 (3.9-4.2)	0.242	4.2 (4.0-4.4)	0.219	3.6 (3.0-4.2)	0.823
	Al Rass	4.1 (4.0-4.3)		4.2 (3.8-4.8)		3.6 (3.1-4.4)	
	Al Bukayriyah	3.85 (3.2-4.2)		3.9 (3.1-4.1)		3.5 (3.0-4.0)	
	Unaizah	4.0 (3.6-4.25)		4.2 (4.0-4.6)		3.6 (3.0-4.0)	

The median (interquartile range) knowledge, attitude, and practice scores of the participants were 4.1 (3.8; 4.3), 4.2 (4.0; 4.6), and 3.6 (3.0; 4.0), respectively. Staff aged 40 years and older had significantly higher scores for KAP than other age-groups. Doctors had significantly higher knowledge and attitude scores than nurses. The score difference between male and female PHC staff was not significantly different. The PHC staff of the four cities had similar levels of KAP.

The participants’ responses about the current leading source of knowledge and preferred source of knowledge about vision and eye screening in preschool children are given in Table 2.

**Table 2:** Source of information (current vs desired) about vision and eye screening of preschool children among PHC staff in Qassim, Saudi Arabia PHC, primary health center

	Current source	Desired source
	Number (percentage)	Number (percentage)
During medical education	52 (51.5)	0 (0.0)
Medical journals	0 (0.0)	26 (25.7)
Training in eye care	2 (2.0)	23 (22.8)
Eye care professionals	12 (11.9)	34 (33.7)
Google and computer	13 (12.9)	4 (4.0)
Social media	15 (14.9)	4 (4.0)
Television	2 (2.0)	2 (2.0)
From feedback of referred patients	2 (2.0)	7 (6.9)
Other	2 (2.0)	0 (0.0)
Pamphlets & brochures	1 (1.0)	1 (1.0)

The primary source of information about PVS was medical education (51%). Other sources were eye care professionals (11.9%), Google and computers (12.9%), and social media (14.9%). The participants’ preferred sources of information were eye professionals (33.7%), medical journals (25.7%), and eye care training (22.8%).

## Discussion

The doctors and nurses of the PHCs in Qassim have good knowledge and positive attitudes toward the eye and vision screening of preschool children. However, practices related to this issue were lower than desired, and many participants were unsure of their practice-related responses. Older staff and doctors had better knowledge and attitudes than other age-groups and female participants. Half of the participants’ knowledge of the topic was based on information they received during their medical education. Google and social media were also partly responsible for their knowledge. Their desired sources of knowledge included medical journals, eye care professionals, and training workshops.

Primary health staff are vital stakeholders in imparting awareness about eye health, detecting diseases in children, and providing timely referrals to proper professionals by counseling parents and arranging appointments with pediatric ophthalmology clinics. The present study identified the status of KAP among PHC staff and their expectations to improve KAP through training, feedback from eye care professionals, and medical journals. On this basis, national and provincial health programs for child health care, primary health care, and eye health care could organize training, as in the neighboring country of Oman [19], and they could provide referrals from primary care to higher levels of eye care and feedback to PHCs [20].

In the present study, the participants’ knowledge level on 10 questions related to vision and eye screening was high. Feedback on identifying amblyopia in preschoolers through vision screening only reflected the weakest component. This contrasted with the findings of Marsh-Tootle et al. [10], who reviewed pediatricians’ and family physicians’ knowledge and PVS behavior and noted a positive association between better knowledge and good PVS behavior. A study in Jordan surveyed 48 pediatricians and found that their knowledge and attitude toward eye screening of children for refractive error, amblyopia, and strabismus were good; however, there were fewer referrals to ophthalmologists than desired [11]. In a study in Kenya that surveyed 125 pediatricians, 70% of participants had poor knowledge about eye diseases in children [12]. Participants in the present study showed low awareness of the importance of vision screening for detecting asymptomatic eye problems in children below five years old. The importance of compliance with spectacle wearing, as recommended to children by eye doctors, was well understood by PHC health staff. The training of health staff on this topic should note these weaknesses and strengths.

The positive attitude of PHC staff toward vision and eye screening of preschool children is encouraging. Health staff with positive attitudes are more likely to adopt behaviors related to PVS [10]. Although pediatricians had a positive attitude in Jordan, their liaison with ophthalmologists for children’s eye care was not good [11]. Knowledge about different components of vision and eye screening could be improved through lectures and workshops for PHC staff. However, changing attitudes is a challenge. Personal experience, lessons learned during referral, and feedback from ophthalmologists contribute to altering attitudes toward referral, counseling parents, and proactively taking care of children with suspected eye problems.

Health care staff have a crucial role in detecting eye problems in the early stages of childhood. Conditions such as impaired vision, amblyopia, and amblyogenic factors are often difficult to notice. Strabismus, if it is of a minor degree, is often missed. Periodic attachment to eye clinics and practical training workshops could improve their skills and practice. Different health staff are utilized for universal vision screening, including doctors, nurses, ophthalmic assistants, and mid-level eye care professionals specially trained in screening. One of the significant barriers to good practices for vision screening among health staff at PHC is workload [21]. The use of digital devices for rapid vision and eye screening is recommended [22]. The health staff of PHC could be trained in using these devices and providing these tools for their use as the first level of screening and referring those who fail to ophthalmologists/optometrists.

In our study, health staff of older age-groups had significantly better knowledge, positive attitudes, and good practices for vision screening of preschool children compared to younger health staff. The recent decrease in the focus on eye care in medical and nursing training could be one factor, which needs to be confirmed and addressed. Older staff may also be parents and have learned about the importance of vision and eye screening for their children, resulting in age-related differences in KAP.

Doctors at PHCs had better knowledge and attitudes toward PVS than nurses at PHCs. This should be noted when formulating the training material for nurses versus doctors. Doctors of PHCs are responsible for referrals to ophthalmologists and receiving feedback from them about referred children. This could be a reason for the difference in knowledge and attitudes. In many countries, vision screening is the responsibility of nursing staff. In this scenario, it is important to improve the knowledge and attitude of nursing staff for better-quality vision and eye screening of preschoolers [23,24].

We did not find a difference in the level of KAP by the gender of the participants. Most of the nurses in Saudi are women. Visiting KGs by male health staff for health screening is a challenge. Thus, the finding that women have similar levels of KAP as men is promising if universal vision and eye screening are implemented in the study area in the coming years.

To some extent, the current sources of information for PHC staff are worrisome. Overuse of social media and unconfirmed sources of cyber knowledge may provide misinformation to health staff. Officially approved sources of information on preschool vision and eye screening could be part of the Ministry of Health’s knowledge portal. Providing standard operating procedure manuals for eye care at PHCs could be helpful guides for health staff [25].

Our study has a few limitations. This was a cross-sectional study with a sample that was not recruited randomly, so extrapolation of the outcomes to the entire health staff of the PHCs in Qassim Province should be carried out with caution.

## Conclusions

The reasonably good level of knowledge, positive attitude, and perceived less healthy practices in the present study suggest the need to strengthen the health staff’s capacity through training, providing resources, and improving the channel of communication between eye care professionals and primary health staff in the catchment areas. The proposed mode of imparting health awareness messages to health staff should be reviewed by administrators. A follow-up survey is recommended to understand the impact of both PVS programs in the study area and the training of health staff in primary eye care, including national eye and vision screening programs.

## Appendices

**Table 3:** Questions about preschool vision and eye screening for primary health center staff

Knowledge	
K1	Less vision in <5 years old children can cause permanent vision impairment/blindness
K2	Less vision in <5 years old children can cause crossed eye
K3	Impaired vision in <5 years old children will need spectacles
K4	Less vision in <5 years old children can affect their development
K5	Less vision in <5 years old children can be detected only by vision testing
K6	<5 years old children with a refractive error should wear spectacles
K7	Children of parents with a refractive error have more chances of having a refractive error
K8	If there is a refractive error in a child in the family, other siblings have more chances of having a refractive error
K9	<5 years old children should be periodically examined by an optometrist/ophthalmologist
K10	Parents should strictly follow "screen time" norms in their children before school age
Attitude	
A1	I believe spectacles should not be used by young children as it increases number of spectacles faster
A2	I believe excessive exposure to computers and phones results in a refractive error in children
A3	I believe parents should not allow children to undergo surgery for cross-eye in young children
A4	I believe children are negatively psychologically affected and therefore should not be given spectacles
A5	I believe <5 years old children should be screened for eye and vision
Practice	
P1	I have taken my <5 years old children to an optician for vision and eye check-up
P2	I have taken my <5 years old children to an ophthalmologist for vision and eye check-up
P3	I have purchased spectacles for my kindergarten child
P4	I make sure that my own less than six years old children use spectacles as per the advice of the eye professional
P5	I strictly follow guidelines of "screen time" for my own less than six years old children
